# Planning for future COVID-19 vaccine procurement

**DOI:** 10.2471/BLT.22.288729

**Published:** 2022-09-01

**Authors:** Yot Teerawattananon, Siobhan Botwright, Murat Hakan Ozturk

**Affiliations:** a Health Intervention and Technology Assessment Program, 6th Floor, 6th Building, Department of Health, Ministry of Public Health, Tiwanon Rd., Muang, Nonthaburi 11000, Thailand.; b Revolving Fund for Access to Vaccines, World Health Organization Regional Office for the Americas, Washington DC, United States of America.

Even before the efficacy trial results of the first coronavirus disease 2019 (COVID-19) vaccine candidates were released, many governments had already made legally binding deals with vaccine manufacturers.^1^ At the beginning of 2021, most countries had secured sufficient doses to vaccinate priority populations.^1^ However, manufacturers were unable to live up to their optimistic supply forecasts. The rushed COVID-19 vaccine development and scale-up, combined with complex regulatory processes and supply chains, led to significant delays in vaccine production and vaccine wastage.^2^ Manufacturers first granted vaccine access for countries that had invested in vaccine research and development and had initiated deals early. In countries experiencing severe delivery delays, supplier dominance meant that governments could not effectively hold manufacturers accountable for timely delivery. Rising public frustration, often coupled with mounting COVID-19 cases, pushed many governments to over-procure despite existing volume commitments with suppliers, in an attempt to ensure timely vaccination of their population.

When vaccines did become available, short expiry dates, fragmented deployment of multiple vaccines, delays in operational funding and shortages of syringes complicated roll-out.^3^^,^^4^ Although global organizations advocated for countries to leverage COVID-19 vaccination for long-term systems strengthening, the emergency nature of the pandemic meant that most governments planned for emergency campaigns, with a surge capacity for human resources to deliver vaccines.^4^^,^^5^ However, because countries did not receive the promised volumes of vaccines on time, many experienced prolonged and unsustainable vaccination campaigns and personnel burn-out.^4^

When manufacturers were able to redress the supply issue, governments received more doses than they could use, which overwhelmed health systems. In all cases, governments had limited flexibility to renegotiate volumes or deliveries, in part due to supplier dominance during the contract negotiation stage.

While many governments have procured more vaccines than they need, others have not yet vaccinated most of their population with the primary series (for most vaccines, two doses). Addressing this issue by creating a mechanism for global vaccine exchange would be an effective and rational action. However, the vaccine demand necessary to sustain such an exchange mechanism is lacking,^6^ since countries with low vaccine coverage are currently not requesting sufficient volumes of vaccines. Stockpiling vaccines for future need is not a viable option, given the short expiry date of COVID-19 vaccines. 

In this situation of oversupply, governments should be cautious of implementing reactive policies solely aimed at curbing wastage. Policies to vaccinate children or to provide multiple booster doses require careful consideration of current evidence and benefit–risk analysis.^7^^,^^8^ Beyond health and cost implications, governments should also consider any consequences on public support for vaccination programmes, including issues of vaccination fatigue or scepticism.

Most countries are not forecasting demand for future COVID-19 vaccines. To protect high-risk groups, governments must look beyond current wastage issues and make COVID-19 vaccine procurement predictable. In countries with sufficient modelling capacity, epidemiological models can inform vaccination strategies. For others, simple rules of thumb can be applied and adjusted, such as procuring booster dose vaccines to cover 20% of the population, or the equivalent to high-risk groups. Without such planning, manufacturers might scale down production and be unable to respond to future demand surges.

As the first vaccines were purchased, scant evidence existed on real-world vaccine effectiveness or future needs related to the pandemic. Governments did not follow conventional decision-making processes for vaccine procurement and vaccination policies, often taking decisions through high-level emergency committees. While such processes have sometimes facilitated the link between modellers and policy-makers, established participatory structures for evidence-based decisions have often not been used. Considerable evidence on the impact of vaccination is now being gathered; therefore, governments need to adopt a transparent, timely and accountable decision-making process. Ideally, this process should extend beyond the current COVID-19 pandemic and boost processes for reliable decision-making in future public health emergencies.

Furthermore, governments should plan to integrate vaccination campaigns with routine health services. One of the main bottlenecks for COVID-19 vaccination in low- and middle-income countries has been the lack of experience in vaccinating adult populations. Experience has shown that intensive vaccination campaigns cannot be sustained over time.^4^ While global health agencies have advocated for countries to use COVID-19 vaccination as an opportunity to build adult vaccination platforms, this approach may not be appealing to countries that do not prioritize resource allocation to adult vaccination. However, all countries have committed to the United Nations declaration on universal health coverage (UHC).^9^

Integrating COVID-19 vaccination with UHC efforts to reach all socioeconomic groups is essential to maintain vaccination campaigns. Stabilizing COVID-19 vaccine supply and delivery will require governments to plan future vaccine needs through accountable processes while strengthening routine systems to deliver the vaccine over the long term.
